# A prenatal acoustic signal of heat reduces a biomarker of chronic stress at adulthood across seasons

**DOI:** 10.3389/fphys.2024.1348993

**Published:** 2024-03-29

**Authors:** Eve Udino, Marja A. Oscos-Snowball, Katherine L. Buchanan, Mylene M. Mariette

**Affiliations:** ^1^ School of Life and Environmental Sciences, Deakin University, Geelong, VIC, Australia; ^2^ Max Planck Institute for Biological Intelligence, Seewiesen, Germany; ^3^ Faculty of Veterinary and Agricultural Sciences, The University of Melbourne, Werribee, VIC, Australia; ^4^ Doñana Biological Station (EBD-CSIC), Sevilla, Spain

**Keywords:** environmental matching hypothesis, heterophil:lymphocyte ratio, leucocyte profile, phenotypic plasticity, *Taeniopygia guttata castanotis*, thermal adaptation

## Abstract

During development, phenotype can be adaptively modulated by environmental conditions, sometimes in the long-term. However, with weather variability increasing under climate change, the potential for maladaptive long-term responses to environmental variations may increase. In the arid-adapted zebra finch, parents emit “heat-calls” when experiencing heat during incubation, which adaptively affects offspring growth in the heat, and adult heat tolerance. This suggests that heat-call exposure may adjust individual phenotype to hot conditions, potentially compromising individual sensitivity to cool weather conditions. To test this hypothesis, we manipulated individual prenatal acoustic and postnatal thermal experiences during development, and sought to assess subsequent chronic responses to thermal fluctuations at adulthood. We thus measured heterophil to lymphocyte (H/L) ratios in adults, when held in outdoor aviaries during two summers and two winters. We found that birds exposed to heat-calls as embryos, had consistently lower H/L ratios than controls at adulthood, indicative of lower chronic stress, irrespective of the season. Nonetheless, in all birds, the H/L ratio did vary with short-term weather fluctuations (2, 5 or 7 days), increasing at more extreme (low and high) air temperatures. In addition, the H/L ratio was higher in males than females. Overall, while H/L ratio may reflect how individuals were being impacted by temperature, heat-call exposed individuals did not show a stronger chronic response in winter, and instead appeared more resilient to thermal variability than control individuals. Our findings therefore suggest that heat-call exposure did not compromise individual sensitivity to low temperatures at adulthood. Our study also reveals that prenatal sound can lead to long-term differences in individual physiology or quality/condition, as reflected by H/L ratios, which are consistent with previously-demonstrated reproductive fitness differences.

## 1 Introduction

Early-life experience can shape the phenotype of individuals in the long-term, by altering developmental trajectories (Lindström, 1999; Bateson et al., 2004). This process, referred to as “developmental programming,” may allow developing individuals to match their phenotypes to future predictable environments. However, when early environmental cues poorly forecast future environments, long-term developmental programming can be maladaptive (Bateson et al., 2004; Gluckman et al., 2005; Monaghan, 2008). Especially for thermal environments, early conditions may not predict those in later-life, particularly under climate change, which increases weather variability and the frequency of extreme weather events (IPCC, 2021). However, whether developmentally plastic traits persist into adulthood or are reversible remains unclear (Burggren, 2020), especially for traits related to thermal physiology (Angilletta, 2009; Tattersall et al., 2012). Notably, in ectotherms, while there are clearly cases of early-life thermal environment affecting long-term thermal preferences or tolerance (e.g., reptiles: Refsnider et al., 2019; flies; Kellermann et al., 2017; fish; Schaefer and Ryan, 2006), meta-analyses report little effect overall across ectotherm taxa (fishes, amphibians, reptiles, invertebrates: Pottier et al., 2022; reptiles; Zhang et al., 2023). In endotherms however, including birds, much less is known about the impact of early-life experience on individual thermal physiology (e.g., thermoregulation capacities, body temperature) later in life, especially at adulthood (Nord and Giroud, 2020; but see Pessato et al., 2022). Yet, in these species, even though prenatal temperatures are typically under parental control, thermal fluctuations still occur through variation in parental behaviour or body temperature (Nord and Giroud, 2020), in addition to fluctuations experienced by offspring postnatally. Studies in poultry suggest that incubation temperature may sometimes influence juvenile body temperature or heat-tolerance (e.g., Piestun et al., 2008a; Collin et al., 2007 reviewed in Loyau et al., 2015), but studies in non-domesticated birds are lacking (Nord and Giroud, 2020). We therefore know little about how developmental history affects the vulnerability of individuals to weather fluctuations later in life. This hinders our capacity to accurately forecast the impact of climate change on avian population health.

Recent evidence highlights that information about the surrounding environment can be transmitted to embryos via sound (Mariette et al., 2021). In the zebra finch (*Taeniopygia castanotis*), an arid-adapted passerine, parents incubating at high temperatures (>29°C–32°C in the nest) produce “heat-calls” (Mariette and Buchanan, 2016; Mariette et al., 2018), through “vocal panting,” an intense form of panting that improves the emitter’s thermoregulation in the heat, in addition to its signalling function to embryos (Mariette and Buchanan, 2016; Pessato et al., 2020). Prenatal exposure to heat-calls adaptively reduces nestling growth in hot nests (Mariette and Buchanan, 2016), possibly through the modulation of their mitochondrial function (Udino et al., 2021). Both effects plausibly contribute to the higher reproductive success of heat-call birds in adulthood (Mariette and Buchanan, 2016), through a reduction of oxidative damage during development. In addition, individuals exposed prenatally to heat-calls have, in adulthood, hotter thermal preferences, and a different panting strategy on hot summer days compared to control individuals (Mariette and Buchanan, 2016; Udino and Mariette, 2022), as well as greater heat tolerance (Pessato et al., 2022). These findings suggest that prenatal heat-calls program embryos for high postnatal temperatures, which is currently the only known transgenerational mechanism for heat adaptation in endotherms (Mariette and Buchanan, 2016). However, by adjusting their phenotype for hot environments, heat-call exposed individuals may be more challenged by cooler temperatures brought on by seasonal fluctuations (e.g., winter).

Individuals’ ability to cope with environmental fluctuations, such as weather variation, can be indicated via stress biomarkers (Krams et al., 2011; Moagi et al., 2021; Skwarska et al., 2022). In particular, the chronic impact of environmental perturbations can be assessed with leucocyte profiles (Davis et al., 2008; Romero and Wingfield, 2015; Skwarska, 2019), as chronic stress alters the relative proportion of different circulating white blood cells (Gross and Siegel, 1983; Davis and Maney, 2018). Among leucocytes, heterophils (in birds; or neutrophils in mammals) and lymphocytes are the most abundant cell types (c.a. 80%), involved in innate and adaptive immunity, respectively (Davis et al., 2008; Campbell, 2015). Under challenging environmental conditions, the heterophil to lymphocyte (H/L) ratio increases in peripheral blood. This mostly results from a concomitant increase of heterophils, and decrease of lymphocytes that are redistributed across body compartments to prepare the body to cope with infection (Gross and Siegel, 1983; Dhabhar, 2002). In birds, even though responses to temperature have mostly been studied in poultry, the H/L ratio has been shown to increase following heat exposure, for hours or weeks (Altan et al., 2003; Prieto and Campo, 2010; Xie et al., 2017), or after a cold spell of one or 2 weeks (Krams et al., 2011). In addition to its usefulness as a chronic stress biomarker, the H/L ratio may be indicative of individual overall health status or “quality”/condition, if higher quality individuals are generally more resilient to environmental challenges (Hylton et al., 2006; Krams et al., 2011). Accordingly, the H/L ratio correlates with adult body mass in several avian species (Gladbach et al., 2010; Włodarczyk et al., 2018; Skwarska, 2019; but see Powell et al., 2013), or even with song repertoire size in song sparrows (*Melospiza melodia*; Pfaff et al., 2007). Ultimately, the H/L ratio has also been reported to predict survival, in both adults (Kilgas et al., 2006; Maness et al., 2023) and young (Hylton et al., 2006) in wild birds.

In this study, we investigated whether early-life conditions affect individual chronic responses to weather variation at adulthood. To this aim, zebra finch embryos were exposed to heat-calls or control-calls, and were subsequently raised under contrasting postnatal nest temperatures as nestlings. At adulthood, we assessed individual H/L ratio in the peripheral bloodstream, over two summers and two late winters, while individuals were held in outdoor aviaries. We predicted that in summer, individuals exposed to prenatal heat-calls and/or reared in hot nests would have lower H/L ratios (i.e., will be less impacted) compared to individuals exposed to control-calls or reared in cooler nests. However, if tailoring phenotype to hot conditions in early-life is traded-off against resilience to cold conditions throughout life, we expected these individuals to have higher H/L ratios (i.e., will be more challenged) in late winter. Alternatively, if heat-call exposure reduces the physiological costs of growing in the heat in a way that improves individual fitness at adulthood (Mariette and Buchanan, 2016), this may be reflected by a consistently lower H/L ratio in heat-call birds, across environmental conditions. If so, the H/L ratio may also positively correlate with individual body mass and be repeatable within individuals. In addition, we verified that the H/L ratio is indeed responsive to seasonal or weather conditions, and also tested which short-term temporal scale (2, 5, or 7 days) and air temperature variable (daily minimum or maximum) best explained variation in this biomarker. We expected individuals to be more strongly impacted (i.e., higher H/L ratio) by thermal extremes (low and high) than mild conditions (i.e., non-linear quadratic temperature effect).

## 2 Material and methods

### 2.1 Experimental subjects

The experiment was carried out at Deakin University (Geelong, Australia), from September 2017 to September 2019. We worked on 51 adult wild-derived zebra finches (27 males, 24 females) of similar age, hatched in captivity and 8–10th generation descendants from a wild population from Northern Victoria. Individuals from both prenatal acoustic treatment groups were housed together in mixed-sex outdoor aviaries, although there were more males than females, in both playback groups. The birds were provided with food (finch seed mix (Golden Cob™ finch mix), grit, cuttlefish bone, fresh greens) and water *ad libitum.* Birds were not breeding at the time of sampling, in either season.

### 2.2 Developmental conditions: prenatal playback and nest temperature

Early-life conditions of the birds were experimentally manipulated as part of a previous playback experiment conducted in 2014 (Mariette and Buchanan, 2016). Eggs were collected from the nests on laying day, replaced with dummy eggs, and placed in a main incubator (Octagon 20 plus, Brinsea, Australia) set at 37.5°C and 60% humidity. On the 10th day of incubation, eggs were transferred to one of the two experimental incubators, broadcasting a playback of either contact calls (control), or heat-calls (treatment) for the last 4–5 days of incubation. Both playbacks also included whine calls, to ensure normal stimulation of the auditory system by calls with a complex acoustic structure. All call types are naturally produced by parents in the nest, to communicate with their partner (contact and whine calls) or when experiencing heat (heat-calls). The playbacks were broadcast inside the incubators via two speakers (Sennheiser HD439) externally connected to an amplifier (Digitech 18W) and an audio player (Zoom H4nSP), and were played daily (9:30 to 18:00; 16 min of control- or heat-calls per hour) until hatching. To prevent any incubator-specific effect, eggs and sound cards were swapped daily between the two experimental incubators.

At hatching, nestlings were identified (by clipping head down feathers) and returned to their parents or foster parents, in nest boxes placed in outdoor aviaries. The different sun exposure of the nest boxes caused a natural temperature variation between the nest boxes, resulting in a gradient of warm to hot nests (range: 3.15°C–6.17°C above air temperature, T_a_). Nest temperatures were measured using temperature loggers (Maxim Integrated iButton, DS1922L-F5; or Minnow-1.0TH, Senonics). The nest temperature was expressed as the nest temperature above T_a_ (i.e., temperature differential, but thereafter referred to as “nest temperature” for simplicity), calculated as the average difference between the maximum daily nest temperature and maximum daily T_a_ (obtained from the Australian Bureau of Meteorology; BOM) over the nestling rearing period (i.e., hatching to 13 days post-hatch). We only considered this temperature measure because, compared to absolute nest temperature, the nest temperature differential better reflects the thermal rearing environment and better explains nestling development (Mariette and Buchanan, 2016).

### 2.3 Blood sampling

We collected blood samples in late winter (September; see “air temperature” section below) and austral summer (February) across 2 years, when the birds were 3 and 4 years old. The order of winter and summer samplings were reversed between the 2 years to prevent confounding effects of age on seasonal variation. There were four sampling periods: in the first year, sampling occurred on the 21-22-23 September 2017 (N = 43 birds with readable smear) and 01-02 February 2018 (N = 36); and in the second year, on the 08 February 2019 (N = 15) and 10 September 2019 (N = 20). Not all individuals were sampled at each period, due to natural death and to minimise handling time, as birds were caught in a group; and some samples (N = 31) were unsuitable for cell count (see below). Of the 51 birds for which we obtained data, 14 were sampled once, 17 twice, 14 three times and 6 four times, for a total of 114 blood smears. These 51 individuals included 18 males and 15 females from the control playback group, and 9 males and 9 females from the heat-call playback group. For six samples from September 2017, the exact sampling date (within the 3 days of sampling) was unknown, so the weather on the second day of sampling (22-Sep-2017) was used. Excluding these six samples instead did not affect the results.

For each period, birds were captured in a group, held in a wooden box, before successive individual sampling in a random manner. We weighed each individual, and collected blood into heparinised capillary tube after puncturing the brachial vein with a 26-gauge needle. Blood smears were prepared immediately by smearing a drop of blood onto a microscope glass slide to obtain a thin blood film (Campbell, 2015). All blood sampling occurred within 60 min post-capture, except for 9 samples collected within 90 min.

Following blood sampling, blood smears were air-dried and stained with Quick Dip staining (Fronine, Australia). Specifically, the slides were fixed in absolute methanol and successively stained into two staining solutions (eosin-Y and methylene azure B) before being rinsed with water, air-dried and stored in a dark container until analysis.

### 2.4 Heterophil/lymphocyte ratio

Leucocyte counts were performed using a compound microscope (Olympus), at ×1000 magnification using oil immersion. All slides were examined by a veterinary clinical pathologist, blind to individual identity and sampling date. For each slide, the first 100 leucocytes were counted, identifying heterophils, lymphocytes, monocytes, eosinophils and basophils. We then calculated the heterophil to lymphocyte ratio by dividing the heterophil number by lymphocyte number (Gross and Siegel, 1983; Davis et al., 2008). Some smears were not readable due to poor smear quality, including staining issues, cells damaged and smears too thick (N = 31 out of 145 slides, 21%).

### 2.5 Air temperature before sampling at adulthood

We obtained daily atmospheric air temperatures (T_a_) from the Australian Bureau of Meteorology, from the Breakwater Geelong Racecourse station, 6.7 km from the aviaries (station number 87184, latitude: −38.1737, longitude: 144.3765, elevation above sea level: 12.9 m).

First, we considered temperature variations broadly as seasonal variations (i.e., categorical variable: summer or late winter). We conservatively refer to the sampling periods in September as “late winter” because temperatures were about 1.0°C warmer than in the coldest winter months (July-August), and 1.8°C cooler than in early spring (October) (BOM data for 2017 and 2019).

**FIGURE 1:**
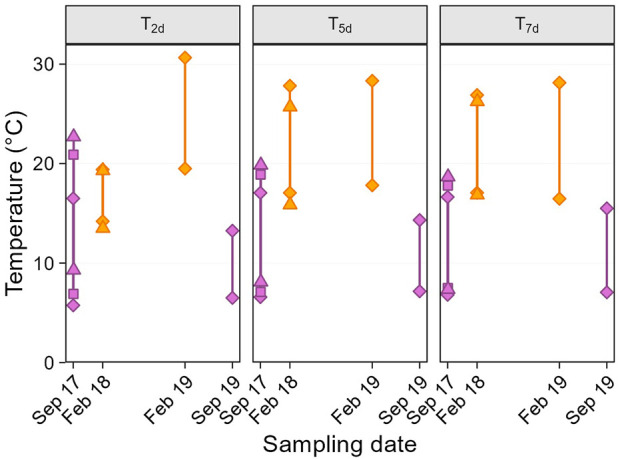
Average maximum and minimum past temperatures before sampling day. Panels show the average of daily temperatures over the 2 (T_2d_), 5 (T_5d_) and 7 (T_7d_) days before sampling. Within panels, upper and lower solid symbols represent average daily maximum and minimum temperatures respectively, and colours indicate the two seasons (purple: late winter, orange: summer). The symbols show the different sampling dates in September 2017 (diamond: 21st, square: 22nd, triangle: 23rd) and February 2018 (diamond: 01^st^, triangle: 02^nd^).

**FIGURE 2:**
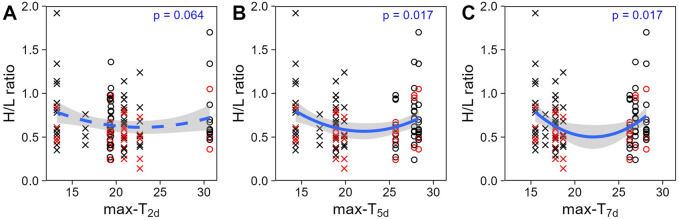
Heterophil to lymphocyte (H/L) ratio (untransformed) across past average maximum temperatures: **(A)** over the 2 days (T_2d_), **(B)** 5 days (T_5d_) or **(C)** 7 days (T_7d_) before sampling day. Colours display the prenatal playback (black = control-calls, red = heat-calls) and symbols the season (cross = late winter, circle = summer). Heat-call bird response to short term weather variation did not differ from control. Overall regression lines are shown with the 95% CI. N = 114 samples from 51 birds.

Second, we considered temperature variation on shorter temporal scales than the seasons, using several “weather variables.” From T_a_ data, we calculated the average daily maximum and minimum temperatures prior to the sampling day, over different time scales (Figure 1): i.e., for 2, 5, and 7 days before sampling (thereafter referred to as max-T_xd_ and min-T_xd_). Longer time scales (e.g., 14 or 30 days, were not considered because the temperature distribution across sampling dates was not suitable for testing quadratic effects (i.e., no middle value, Figure 2). In addition, to specifically focus on temperature variability, we defined a predictor called “weather trend” (two-level categorical variable: warming or cooling), indicating whether the weather in the 2 days just before sampling was warmer or cooler than in the 5 days prior (i.e., period from day −3 to −7).

### 2.6 Statistical analyses

All analyses were performed in R (version 4.0.1).

First, we tested whether the H/L ratio was affected by developmental conditions, season (summer or late winter), and sex. We ran linear mixed models (LMMs) including the H/L ratio as the response variable, the prenatal playback, postnatal nest temperature, season and sex as fixed effects, as well as the two-way interactions between season and the playback or nest temperature.

Second, to test for effects at shorter temporal scales than the seasons, we used a similar approach but replacing the categorical variable “season” by a short-term weather predictor. We ran four separate LMMs, corresponding to the four different weather variables: average maximal temperature over 2, 5, or 7 days before blood sampling or “weather trend” (two-level factor, see above). All models included the H/L ratio as the response variable, prenatal playback, postnatal nest temperature, sex, a weather variable (max-T_2d_, or max-T_5d_, max-T_7d_, or weather trend) as main effects, as well as the two-way interactions between the weather variable and the playback or the nest temperature. Non-significant predictors (*p* > 0.060) were sequentially dropped to obtain a reduced model. Then, we compared the Akaike Information Criterions corrected for small sample size (AICcs) of the three reduced models varying only by the continuous weather predictor to identify which temporal scale(s) best explained variation in H/L ratio, using the *MuMin* package (Bartoń, 2022). Average maximum temperatures were strongly correlated to minimum temperatures: Spearman correlation tests: T_2d_: r_s_ = 0.55, *p* < 0.001; T_5d_: r_s_ = 0.87, *p* < 0.001; T_7d_: r_s_ = 0.82, *p* < 0.001. Nonetheless, to test which of the two best explain variation in H/L ratios, we ran another three LMMs, where the average maximum daily temperature for T_2d_, T_5d_ or T_7d_ was replaced by the corresponding minimum temperature value in the reduced models.

For all models, the *lme4* and*, lmerTest* packages were used (Kuznetsova et al., 2017; Bates et al., 2021), the H/L ratio was log-transformed, continuous predictors were scaled (i.e., mean = 0, std = 1) and bird-ID was included as random factor. Normality of the residuals of full models was visually inspected. Full models are presented in the (Supplementary Tables S1, S2). Given that the H/L ratio does not significantly increase within 60 min of restraint time, but may do so past 60 min (Davis, 2005; Cirule et al., 2012), we re-ran all analyses excluding the nine samples collected >60 min post-capture. Results were qualitatively unchanged, unless otherwise specified.

Third, to probe whether H/L ratio could be indicative of individual health status, quality or condition, we investigated the relationship between body mass and H/L ratio. To obtain data comparable across sampling periods, both the H/L ratio and body mass were standardized (using the *scale* function; mean = 0, standard deviation = 1) within each of the four sampling periods, following (Ochs and Dawson, 2008). We ran a LMM fitting the H/L ratio as response and body mass as predictor. In addition, we evaluated the overall repeatability (across all sampling periods) of individual body mass and H/L ratio by calculating intra-class correlations (ICCs) using the *rptR* package and a Gaussian distribution (Stoffel et al., 2017). Specifically, the scaled body mass or the H/L ratio was fitted as the response variable, and bird identity (“bird-ID”) as the random factor. The 95% confidence intervals were determined by 1000 bootstrap iterations and *p*-values obtained from likelihood ratio tests (LRTs). To test whether repeatability remained after controlling for individual-specific traits and other (significant) fixed effects, we assess “adjusted repeatability” (*sensu* Nakagawa and Schielzeth, 2010), using the same test but controlling for playback, sex and max-T_5d_ as fixed effects. Further, to test specifically whether the trade-off between hot and cold weather tolerance affected heat-call individuals more than control-call individuals, we tested the within-individual repeatability in H/L ratio across seasons (i.e., winter vs*.* summer: Sept vs*.* Feb) using one Pearson’s correlation test per playback group.

## 3 Results

**FIGURE 3:**
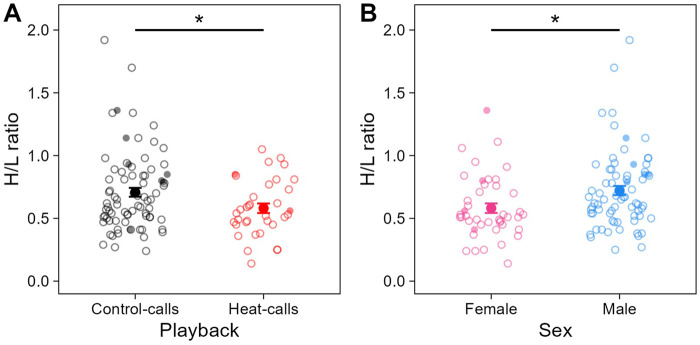
Heterophil to lymphocyte (H/L) ratio across **(A)** prenatal playbacks (black: control-calls, N = 79 samples from 33 birds; red: heat-calls, N = 35 samples from 18 birds) and **(B)** sex (pink: females, N = 43 samples from 24 birds; blue: males, N = 71 samples from 27 birds). Small circles (open or solid) show raw individual data, and larger solid circles show means (±SE). When excluding the nine samples collected >60 min post-capture (small solid circles), the effect of playback felt just below significance (LMM: estimate = −0.19, se = 0.10, t = −1.94, *p* = 0.058). *p < 0.05.

Individuals’ prenatal acoustic experience affected their H/L ratio at adulthood, three to 4 years later. Specifically, the H/L ratio was significantly lower in birds that were prenatally exposed to heat-calls, compared to those exposed to control-calls (Table 1; Figure 3A). This was the case in summer, as predicted, but also in late winter (i.e., no interaction playback x season, Supplementary Table S1), and H/L ratios did not differ overall between seasons. By contrast to prenatal playback, nest temperature experienced postnatally had no effect on H/L ratios at adulthood, in either season (Supplementary Table S1). Lastly, the H/L ratio also differed between the sexes, being significantly higher in males than females (Table 1; Figure 3B).

**TABLE 1:** Reduced linear mixed model^a^ fitting the heterophil to lymphocyte ratio (log-transformed) as a response to developmental conditions, sex and season. Est. = estimates, SE = standard error. N = 114 samples from 51 individuals. When excluding the nine samples collected >60 min post-capture, the effect of playback was marginal (LMM: estimate = −0.19, se = 0.10, t = −1.94, *p* = 0.058).

Predictor	Est	SE	t value	*p*-value
Intercept	−0.56	0.07	−7.52	<0.001
**playback (heat-call)**	−0.20	0.09	2.14	**0.037**
**sex (male)**	0.21	0.09	2.42	**0.019**

^a^ Full model: H/L ratio ∼ playback + nest temperature + sex + season + playback x season + nest temperature x season + (1|bird-ID).

Bold values indicate significant effects.

Unlike at the seasonal scale, the weather experienced in the week prior to blood sampling did affect H/L ratios. H/L ratios were higher at the colder and hotter extremes, compared to milder temperatures (i.e., quadratic effect, Table 2). When comparing time scales, max-T_5d_ and max-T_7d_ were equivalent (△AIC <1, Table 3), and better explained variation in H/L ratio than did max-T_2d_ (△AIC >3, Table 3), in agreement with max-T_2d_ having only a marginal quadratic effect on H/L ratios (whereas max-T_5d_ and max-T_7d_ had a significant effect). In addition, average maximum temperature had similar model fits than average minimum temperature (AICcs: T_2d_: max < min, △AICcs = 0.30; T_5d_: min < max, △AICcs = 1.15; T_7d_: max < min, △AICcs = 1.99). However, even on shorter time scales than the season, the effect of prenatal playback on H/L ratio did not vary with the temperature in the previous days, or temperature trend (i.e., no interaction between playback and any of the four weather variables). Nonetheless, adding weather predictors in the model (Table 2) slightly weakened the effect of playback (becoming marginal: 0.059 < *p* < 0.069), which suggests that responses to weather conditions may partly explain some differences between playback groups. Finally, the temperature individuals had experienced during postnatal development also did not explain responses of H/L ratios to weather fluctuations at adulthood (i.e., no interaction between postnatal nest temperature and weather variables; Supplementary Table S2).

**TABLE 2:** Reduced linear mixed models^a^ fitting adult heterophil to lymphocyte ratio (log-transformed) as response to developmental conditions, sex and past average maximum temperatures over two, or five, or 7 days prior to blood sampling. Est. = estimates, SE = standard error. N = 114 samples from 51 individuals. Results remain similar when excluding the nine samples collected more than 60 min post-capture, except for (max-T_2d_)^2^ in model 1 becoming significant (*p* = 0.035).

Model	Predictor	Est	SE	t value	*p*-value
1. Max-T_2_					
	Intercept	0.52	0.53	0.99	0.326
	playback (heat-call)	−0.18	0.09	−1.93	0.059
	**sex (male)**	0.20	0.09	2.21	**0.032**
	**max-T**_**2d**_	−0.10	0.05	−2.00	**0.049**
	(max-T_2d_)^2^	0.00	0.00	1.88	0.064
2. Max-T_5_					
	Intercept	1.63	0.87	1.88	0.064
	playback (heat-call)	−0.17	0.09	−1.86	0.069
	**sex (male)**	0.20	0.09	2.28	**0.027**
	**max-T**_**5d**_	−0.21	0.08	−2.49	**0.015**
	**(max-T**_**5d**_ **)** ^**2**^	0.00	0.00	2.43	**0.017**
3. Max-T_7_					
	Intercept	3.24	1.53	2.11	0.037
	playback (heat-call)	−0.18	0.09	−1.92	0.060
	**sex (male)**	0.19	0.09	2.20	**0.033**
	**max-T**_**7d**_	−0.36	0.15	−2.46	**0.016**
	**(max-T**_**7d**_ **)** ^**2**^	0.01	0.00	2.43	**0.017**

^a^ Full models: H/L ratio ∼ playback + nest temperature + sex + max-T_xd_ + (max-T_xd_)2 + playback x max-T_xd_ + nest temperature x max-T_xd_ + (1|bird-ID).

Bold values indicate significant effects.

**TABLE 3:** Comparison of AICcs from reduced models (Table 2) testing the effect of past average maximum temperatures on the heterophil to lymphocyte ratio. N = 114 samples from 51 individuals.

Model	Log-likelihood	AICc	△AICc	Weight
3. Max-T_7_	−68.9	152.9	0.00	0.565
2. Max-T_5_	−69.4	153.8	0.93	0.356
1. Max-T_2_	−70.9	156.8	3.92	0.079

**FIGURE 4:**
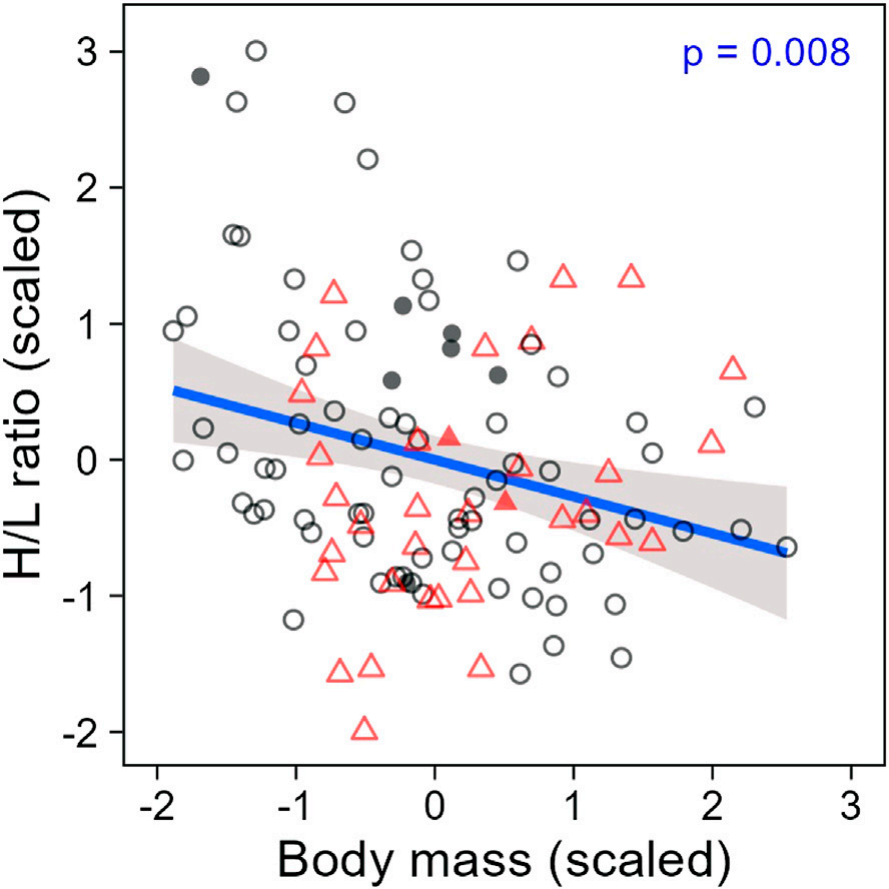
Heterophil to lymphocyte ratio along body mass in individuals prenatally exposed to control calls (black circles) or heat-calls (red triangles). Open and solid symbols represent scaled individual measurements collected within or after 60 min of waiting time post-capture, respectively. The regression line is shown with the 95% CI. N = 114 measurements from 51 individuals. The regression remains significant when excluding the nine samples collected >60 min (LMM: estimate = −0.22, se = 0.10, t = −2.31, *p* = 0.023).

**FIGURE 5:**
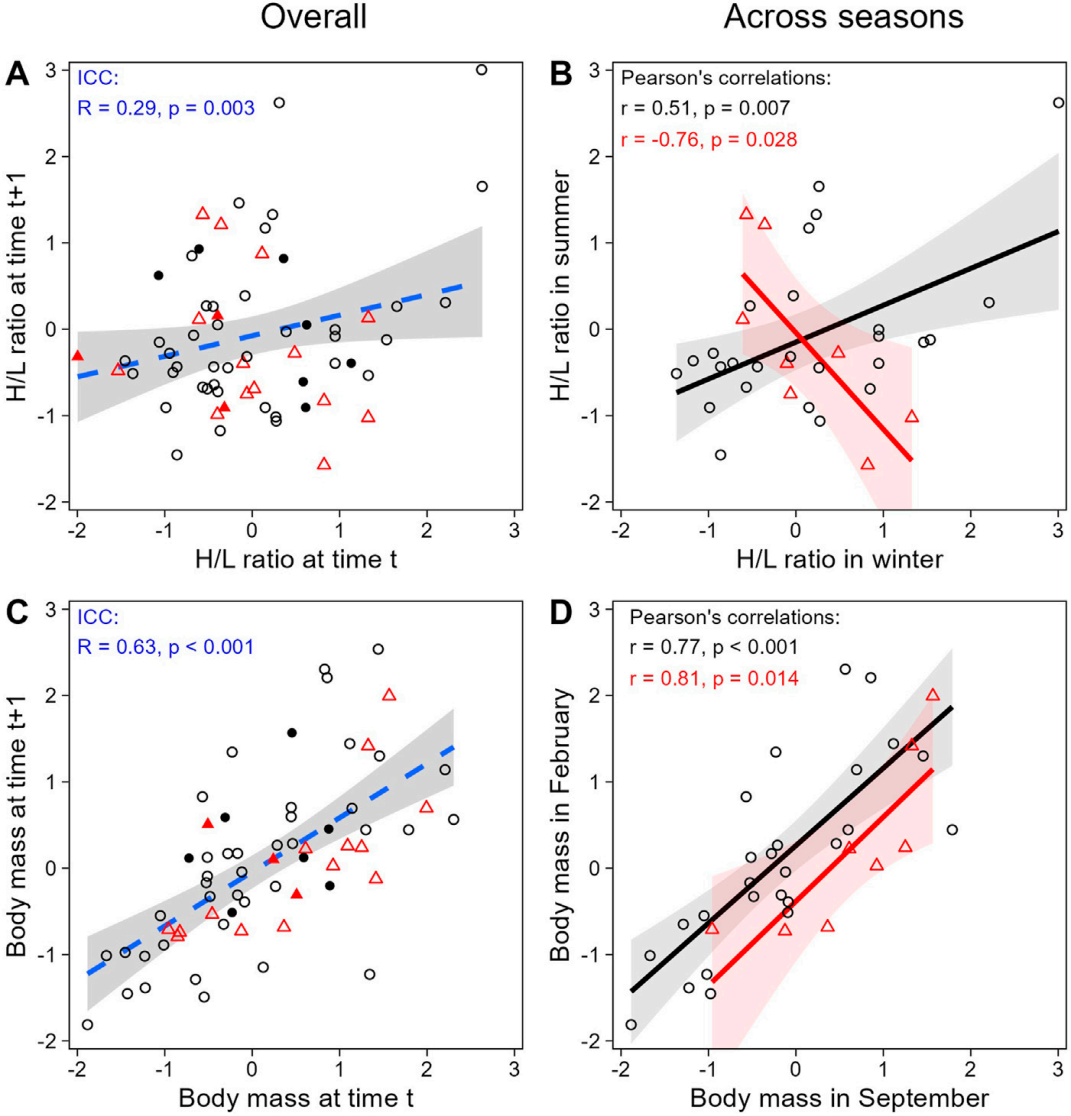
Within-individual repeatability in H/L ratio (top row) and body mass (bottom row), overall across all 4 sampling periods (left column) or across different seasons only (right column). The overall plots **(A and C)** show the repeatability of consecutive measures (i.e., first vs*.* second measures, second vs*.* third, and third vs*.* fourth) and are displayed only for illustration purposes (since some measures are represented twice here, but were considered only once in the ICC analysis). Across seasons **(B and D)**, consecutive sampling dates are compared in winter versus summer (Sep-17 vs*.* Feb-18 + Sep-19 vs*.* Feb-19). Black circles and red triangles represent individual data from control-call and heat-call playback groups, respectively. Open symbols correspond to paired samples both collected below 60 min post-capture; solid symbols are depicted when at least one of the paired samples was collected after 60 min. Data are shown as scaled values. The regression lines are shown with the 95% CI, and dashed lines are for illustration only.

When evaluating H/L ratio as an individual trait, we found that the H/L ratio was negatively correlated to body mass, as expected (LMM; estimate = −0.26, se = 0.10, t = −2.70, *p* = 0.008; Figure 4). In addition, both H/L ratio and body mass were significantly repeatable within individuals across all sampling periods together (ICC; H/L ratio: R = 0.29, se = 0.11, CI = [0.059, 0.504], *p* = 0.003; body mass: R = 0.63, se = 0.08, CI = [0.450, 0.765], *p* < 0.001; Figures 5A,C), even when individuals attributes (sex and playback groups) and weather were accounted for (estimates and significance nearly identical for “adjusted repeatability”; Supplementary Table S3). However, when only considering repeatability across seasons, to identify potential trade-off between responses to hot and cold weather, individual H/L ratios for heat-call birds were negatively correlated in winter vs*.* summer (Pearson’s correlation: *r* = −0.76, *p* = 0.028, N = 8 birds). Control-call birds, by contrast, showed no such trade-off, having a significant positive correlation between winter and summer H/L ratio values (*r* = 0.51, *p* = 0.007, N = 26 birds; Figure 5B). These differences in H/L ratio were not reflected in corresponding changes in body mass, since, in both playback groups, body mass in winter vs*.* summer was positively correlated within individuals (control-calls: *r* = 0.77, *p* < 0.001; heat-calls: *r* = 0.81, *p* = 0.014; Figure 5D).

## 4 Discussion

Our study shows for the first time in any species that prenatal acoustic experience translates into mildly different levels of a biomarker of chronic stress three to 4 years later at adulthood. Specifically, embryonic exposure to heat-calls led to a consistently lower H/L ratio in adult zebra finches, and so in both summer and winter. Nonetheless, H/L ratios did vary with current weather conditions, but only over short-term (five and 7 days), and not between seasons, which likely reflects the acclimatisation capacity of individuals to seasonal fluctuations. Lastly, H/L ratios varied between the sexes, being higher in males than females, and were slightly higher in lighter individuals. Repeatability of H/L ratios within individuals was low although significant, as also found in other avian studies (Norte et al., 2008; Ochs and Dawson, 2008). Altogether, our findings show that prenatal heat-call exposure coincides with lower H/L ratios at adulthood, most likely through carry-over rather than direct effects. This result is consistent with the previously reported positive effect of heat-call exposure on later reproductive success (Mariette and Buchanan, 2016), and points towards a higher resilience to thermal fluctuations in heat-call birds. In any case, our results suggest that heat-call exposure did not make individuals more susceptible to winter conditions over the long-term. This, added to nest temperatures during development having no effect on H/L ratios at adulthood, indicates that early-life experience may not necessarily constrain weather tolerance later in life.

Prenatal acoustic experience affected H/L ratios in adult zebra finches, even though that effect fell just below significance (*p* = 0.058), when nine individuals sampled after 60 min were excluded. H/L ratios levels were lower in both summer and late winter in birds that were prenatally exposed to heat-calls, compared to those exposed to control-calls. Since (short-term) weather conditions did affect H/L ratios, our results may indicate that heat-call exposed birds are less challenged by temperature fluctuations, even towards colder extremes. A lesser impact of high temperatures on heat-call birds is consistent with previous studies showing that heat-call birds have hotter thermal preferences at adulthood and higher heat tolerance (Mariette and Buchanan, 2016; Pessato et al., 2022). However, the lack of any impact of cold conditions on heat-call birds was not necessarily expected. It is possible that late winter temperatures were not low enough to negatively affect heat-call birds, but our study nonetheless unequivocally shows that heat-call bird advantage is not restricted to hot summer conditions. Therefore, it might be that, in addition to preparing individuals for heat, heat-call exposure prepares them for more variable conditions (since in their natural environment, summer rain (accompanied by a cold snap) is often preceded by a heat-wave; M. Mariette, Pers. obs). This may occur via greater physiological flexibility, including of mitochondrial functions, which (in the heat) are more responsive to current thermal environment in heat-call nestlings than in controls (Udino et al., 2021). In addition, heat-call birds could conceivably achieve higher tolerance to temperature fluctuations through a better behavioural thermoregulatory response, as we previously reported in hot weather (Udino and Mariette, 2022). While their thermoregulatory behavioural adjustments to cold conditions (e.g., warm microsite use, huddling behaviour: Chaplin, 1982; Marsh and Dawson, 1989; McKechnie and Lovegrove, 2001) remain to be tested, persistent differences in feeding behaviour at adulthood after exposure to prenatal heat-calls have been demonstrated (Katsis et al., 2021). Alternatively, if H/L ratio reflects individual “quality”/condition (Moreno et al., 2002; Pfaff et al., 2007; Davis et al., 2008), our results support the hypothesis that heat-call exposed individuals had a better somatic state overall at adulthood (Mariette, 2020), possibly underlying their higher reproductive success previously reported (Mariette and Buchanan, 2016). Indeed, it has been hypothesised (but not tested), that, by reducing growth in hot weather, heat-call exposure may reduce oxidative damage during development, leading to higher condition later in life (Mariette and Buchanan, 2016; Mariette, 2020). Consistent with this hypothesis, mitochondrial function of heat-call nestlings shifted towards higher *LEAK* respiration in extreme heat (Udino et al., 2021), which is expected to minimise oxidative damage associated with reactive oxygen species production (uncoupling to survive hypothesis; Brand, 2000). Lastly, the prenatal acoustic experience affected the H/L ratio repeatability across seasons, with the H/L ratio being positively correlated in control-call birds but negatively correlated in heat-call birds. This may indicate that while control birds did not show an apparent trade-off in their response to seasonal temperature variation, heat-call individuals did. Within heat-call individuals, those least affected by heat (i.e., with a higher H/L ratio in summer) were most affected by the cold (i.e., had a lower H/L ratio in winter). However, this result should be confirmed with a larger sample size in future studies.

In contrast to prenatal acoustic experience, the postnatal nest temperature did not affect H/L ratios at adulthood. To date, the long-term effects of early thermal experience on later-life thermoregulation have been surprisingly understudied in endotherms, unlike in ectotherms (Refsnider et al., 2019; Nord and Giroud, 2020; Pottier et al., 2022). Nonetheless, prenatal heat-calls, signalling hot conditions, improved heat tolerance in adult zebra finches (Pessato et al., 2022). In our previous study, nestlings reared in hot nests were then more likely to pant during summer days at adulthood, than those reared in cool nests, although the adaptive value of this effect remains unknown (Udino and Mariette, 2022). In poultry, manipulation of incubation temperatures in chicken *(Gallus domesticus*; 1.7°C above optimum (control) temperature), although detrimental to hatching success, can improve tolerance to a heat-challenge in juveniles (Piestun et al., 2008a; 2008b) but only in some cases (e.g., Collin et al., 2007). As for post-hatch thermal environment, warm rearing conditions (30°C) led to higher bill surface temperatures in 3-month old quail (*Coturnix japonica*) compared to those reared under cold conditions (15°C; Burness et al., 2013).

Surprisingly, despite the well-recognised seasonal modulations of the immune system (Nelson et al., 2002), the effects of seasonal temperature fluctuations on H/L ratio has rarely been investigated (Skwarska, 2019). Yet, our study shows that H/L ratio varied with average daily maximum temperatures in the days prior to measurement. In particular, past temperatures over the five or 7 days before sampling better explained variation in H/L ratio than temperatures over the 2 days before sampling. This could be caused by the cumulative exposure to challenging temperatures over multiple days, resulting in greater impacts on chronic stress levels. Although we could not test the impact of temperature over the past 14 or 30 days in our study, such cumulative exposure was also observed in first-year adult female great tits (*Parus major*), which showed a more pronounced increase of H/L ratio after 2 weeks of cold spell than 1 week (Krams et al., 2011). By contrast, long-term temperature in our study, considered as seasonal variation (as a categorical predictor), did not affect chronic stress levels. This may reflect individual acclimatisation capacity, which reduces sensitivity to long-term climatic variations (Hart, 1962; Somero, 2010; Beaman et al., 2016). Nonetheless, wild zebra finches were recently found to acclimatize their thermoregulation capacities to current weather conditions surprisingly rapidly, adjusting within just 1 day of temperature shift (at least in summer; Pessato et al., 2023). The impact of short-term weather variation on H/L ratio documented here might thus reflect a cost of such plasticity, or stem from rapid acclimatisation being incomplete compared to seasonal acclimatisation. Overall, our study demonstrates the usefulness of H/L ratio for assessing how individuals are coping with temperature fluctuations, which could be particularly relevant for conservation purposes.

Likewise, the repeatability of the H/L ratio has been rarely documented. Here, the H/L ratio was overall weakly although significantly repeatable within individuals kept in outdoor aviaries in non-breeding conditions (prior or during sampling periods). However, H/L ratios were correlated across seasons in opposite directions between the playback groups, suggesting that early-life experience can affect the H/L ratio repeatability at adulthood. Such effects of early-life experience could therefore contribute to the H/L ratio variability among studies, but also to a low repeatability overall. No repeatability in H/L ratios was found across breeding seasons in wild tree swallows (*Tachycineta bicolor*), or within and across years in wild great tits (Norte et al., 2008; Ochs and Dawson, 2008), although it was repeatable over periods of 45 days (Norte et al., 2008). By contrast, the H/L ratio was repeatable over both the short- (4–8 days) and long-term (>4 months) in captive greenfinches (*Carduelis chloris*) housed indoor, under constant conditions (Hõrak et al., 2002). It therefore appears from these studies, that the sensitivity of H/L ratio to environmental conditions can mask intrinsic differences in H/L ratio between individuals. In addition, it is likely that, unlike for traits such as mass, measurement error in H/L ratios also contribute to lower repeatability across measurements.

Finally, we found that H/L ratio is sex-dependent, being higher in males than females. This is despite the two sexes being of similar size in zebra finches, and male zebra finches having higher acclimatisation capacities than females in response to rapid weather fluctuations (Pessato et al., 2022). Sex differences in immune function have often been reported, even though there are no clear explanations for such differences (Forbes, 2007; Hasselquist, 2007). With regard to the H/L ratio, sex differences have sometimes been found, depending on the breeding status of individuals (reviewed in Skwarska, 2019). For instance, the H/L ratio was higher in great tit males during pre-breeding periods, whereas it was higher in females during breeding (Skwarska, 2019). In addition, a recent meta-analysis integrating 41 wild avian species showed that greater elevation of the H/L ratio occurs in males than females between non-breeding and breeding status (Valdebenito et al., 2021). Even though birds were not breeding in our study, as an opportunistic breeder (breeding when conditions become favourable), the zebra finch can remain in a reproductive state in non-breeding periods (Zann, 1996; Perfito et al., 2007). Nonetheless, sex differences in H/L ratio have also been reported among non-breeding birds, including in chicken (greater in males: Campo and Davila, 2002), Pekin ducks (*Anas platyrhynchos domesticus*; greater reactivity to glucocorticoid release in females Tetel et al., 2022) and in great-tits (greater reactivity to social stress in females: Meer and Oers, 2015). Furthermore, the immune system also responds to social stress in humans and other animals (Snyder-Mackler et al., 2020). In great tits, the H/L ratio response to social stress was higher in females, which also experienced more agonistic interactions than males (Meer and Oers, 2015). Zebra finches, nonetheless, are colonial and do not establish group hierarchy (Zann, 1996).

To conclude, our study shows that, in contrast to early thermal experience, prenatal acoustic experience leads to subtle but detectable long-lasting changes on a chronic stress index at adulthood. Individuals exposed to heat-calls prenatally had consistently lower H/L ratios across the natural thermal range tested, and H/L ratio was moderately repeatable within individuals, and correlated to body mass. Furthermore, our results identified that, in contrast to seasons, temperatures experienced over short-term scales affected the H/L ratio. Overall, our findings bring a better understanding of the role of developmental programming and phenotypic plasticity on the impact of weather on population health and of the consequences of prenatal sounds on adult physiology.

## Data Availability

The original contributions presented in the study are included in the article/Supplementary Material, further inquiries can be directed to the corresponding authors.
