# Supplementary material for: A prenatal acoustic signal of heat reduces a biomarker of chronic stress at adulthood across seasons
Source: Front Physiol. 2024 Mar 29;15:1348993. doi: 10.3389/fphys.2024.1348993 (PMC11009423; doi:10.3389/fphys.2024.1348993)
Supplement: Supplementary file 1 [file Table1.DOCX]

Supplementary Material

A prenatal acoustic signal of heat reduces a biomarker of chronic stress at adulthood across seasons

Eve Udino^1,2,*^, Marja A. Oscos-Snowball^3^, Katherine L. Buchanan^1^, Mylene M. Mariette^1,4,*^

^1^School of Life & Environmental Sciences, Deakin University, 75 Pigdons Road, Waurn Ponds VIC 3216, Australia

^2^ Max Planck Institute for Biological Intelligence, Eberhard-Gwinner-Straße, 82319, Seewiesen, Germany

^3^Faculty of Veterinary and Agricultural Sciences, The University of Melbourne, Werribee, VIC, Australia

^4^Doñana Biological Station (EBD-CSIC), Calle Américo-Vespucio 26, 41092 Sevilla, Spain

*** Correspondence:**Corresponding Authors
[eve.udino@bi.mpg.de](mailto:eve.udino@bi.mpg.de), [m.mariette@deakin.edu.au](mailto:udinoe@deakin.edu.au)

# Supplementary Figures and Tables

## Supplementary Tables

**Table S1**. Full linear mixed model^1^ fitting the heterophil to lymphocyte ratio (log-transformed) as a response to developmental conditions, sex and season (N = 114 samples from 51 individuals). Bold values indicate significant effects. Est. = estimates, SE = standard error.

| Predictor | Est. | SE | t value | p-value | random factor [bird-ID] variance (±SE) |
| --- | --- | --- | --- | --- | --- |
| Intercept | -0.59 | 0.09 | -6.68 | <0.001 | 0.02 (±0.16) |
| playback (heat-call) | -0.17 | 0.14 | -1.22 | 0.225 |  |
| nest temperature | -0.05 | 0.06 | -0.78 | 0.438 |  |
| **sex (male)** | 0.22 | 0.09 | 2.47 | **0.017** |  |
| season | 0.04 | 0.09 | 0.46 | 0.648 |  |
| playback x season | -0.05 | 0.16 | -0.33 | 0.740 |  |
| nest temperature x season | 0.02 | 0.08 | 0.25 | 0.804 |  |

^1^Full model: H/L ratio ~ playback + nest temperature + sex + season + playback x season + nest temperature x season + (1|bird-ID)

**Table S2**. Full linear mixed models fitting the heterophil to lymphocyte ratio (log-transformed) as response to developmental conditions, sex and past average maximum temperatures over two, five, or seven days prior to blood sampling, or weather trend. Bold values indicate significant effects. Est. = estimates, SE = standard error. N = 114 samples from 51 individuals.

| Model | Predictor | Est. | SE | t value | p-value | random factor [bird-ID] variance (±SE) |
| --- | --- | --- | --- | --- | --- | --- |
| 1^1^. Max-T_2d_ | Intercept | 0.44 | 0.54 | 0.81 | 0.419 | 0.03 (±0.18) |
|  | playback (heat-call) | 0.02 | 0.37 | 0.06 | 0.953 |  |
|  | nest temperature | -0.08 | 0.22 | -0.37 | 0.710 |  |
|  | **sex (male)** | 0.20 | 0.09 | 2.19 | **0.033** |  |
|  | max-T_2d_ | -0.09 | 0.05 | -1.86 | 0.067 |  |
|  | (max-T_2d_)^2^ | 0.00 | 0.00 | 1.80 | 0.076 |  |
|  | playback x max-T_2d_ | -0.01 | 0.02 | -0.56 | 0.581 |  |
|  | nest temperature x max-T_2d_ | 0.00 | 0.01 | 0.23 | 0.817 |  |
| 2^1^. Max-T_5d_ | Intercept | 1.59 | 0.88 | 1.80 | 0.076 | 0.03 (±0.18) |
|  | playback (heat-call) | -0.16 | 0.36 | -0.45 | 0.656 |  |
|  | nest temperature | 0.04 | 0.18 | 0.21 | 0.834 |  |
|  | **sex (male)** | 0.21 | 0.09 | 2.30 | **0.026** |  |
|  | **max-T_5d_** | -0.20 | 0.09 | -2.40 | **0.019** |  |
|  | **(max-T_5d_)^2^** | 0.00 | 0.00 | 2.34 | **0.022** |  |
|  | playback x max-T_5d_ | 0.00 | 0.02 | -0.04 | 0.970 |  |
|  | nest temperature x max-T_5d_ | 0.00 | 0.01 | -0.37 | 0.710 |  |
| 3^1^. Max-T_7d_ | Intercept | 3.17 | 1.56 | 2.03 | 0.046 | 0.03 (±0.18) |
|  | playback (heat-call) | -0.15 | 0.36 | -0.42 | 0.679 |  |
|  | nest temperature | 0.03 | 0.17 | 0.20 | 0.843 |  |
|  | **sex (male)** | 0.20 | 0.09 | 2.23 | **0.030** |  |
|  | **max-T_7d_** | -0.35 | 0.15 | -2.36 | **0.021** |  |
|  | **(max-T_7d_)^2^** | 0.01 | 0.00 | 2.33 | **0.022** |  |
|  | playback x max-T_7d_ | 0.00 | 0.02 | -0.09 | 0.928 |  |
|  | nest temperature x max-T_7d_ | 0.00 | 0.01 | -0.37 | 0.713 |  |
| 4^2^. Weather trend | Intercept | -0.56 | 0.08 | -6.86 | <0.001 | 0.02 (±0.15) |
|  | playback (heat-call) | -0.14 | 0.13 | -1.11 | 0.272 |  |
|  | nest temperature | -0.06 | 0.06 | -1.07 | 0.287 |  |
|  | sex (male) | 0.22 | 0.09 | -0.36 | 0.721 |  |
|  | weather trend | -0.03 | 0.09 | -0.36 | 0.721 |  |
|  | playback x weather trend | -0.07 | 0.16 | -0.42 | 0.673 |  |
|  | nest temp. x weather trend | 0.05 | 0.07 | 0.64 | 0.527 |  |

^1^Full models: H/L ratio ~ playback + nest temperature + sex + max-T_xd_ + (max-T_xd_)^2^ + playback x max-T_xd_ + nest temperature x max-T_xd_ + (1|bird-ID)

^2^Full model: H/L ratio ~ playback + nest temperature + sex + weather trend + playback x weather trend + nest temperature x weather trend + (1|bird-ID)

**Table S3.** Within-individual **r**epeatability in body mass and heterophil to lymphocyte ratio (scaled per sampling period), while controlling or not for playback, sex and max-T_5d_ as fixed effects (N = 114 samples from 51 individuals). Repeatability of the H/L ratio was also estimated again after excluding scaled values > 2 (N = 109 samples from 50 individuals) and results remain similar. Bold values indicate significant effects. SE = standard error, CI = 95% confidence intervals.

| Control for fixed effects | | no | yes |
| --- | --- | --- | --- |
| Body mass | R | 0.63 | 0.63 |
|  | SE | 0.08 | 0.09 |
|  | CI | [0.450, 0.765] | [0.432, 0.768] |
|  | p-value | **<0.001** | **<0.001** |
| H/L ratio | R | 0.29 | 0.25 |
|  | SE | 0.11 | 0.12 |
|  | CI | [0.059, 0.504] | [0.039, 0.497] |
|  | p-value | **0.003** | **0.012** |
